# Altered gut microbiota and its association with inflammation in patients with chronic thromboembolic pulmonary hypertension: a single-center observational study in Japan

**DOI:** 10.1186/s12890-022-01932-0

**Published:** 2022-04-08

**Authors:** Yumiko Ikubo, Takayuki Jujo Sanada, Koji Hosomi, Jonguk Park, Akira Naito, Hiroki Shoji, Tomoko Misawa, Rika Suda, Ayumi Sekine, Toshihiko Sugiura, Ayako Shigeta, Hinako Nanri, Seiichiro Sakao, Nobuhiro Tanabe, Kenji Mizuguchi, Jun Kunisawa, Takuji Suzuki, Koichiro Tatsumi

**Affiliations:** 1 Department of Respirology, Graduate School of Medicine, Chiba University, 1-8-1, Inohana, Chuo-Ku, Chiba City, 260-8670 Japan; 2 Laboratory of Vaccine Materials, Center for Vaccine and Adjuvant Research and Laboratory of Gut Environmental System, National Institutes of Biomedical Innovation, Health, and Nutrition, Osaka, Japan; 3 Laboratory of Bioinformatics, Artificial Intelligence Center for Health and Biomedical Research, National Institutes of Biomedical Innovation, Health and Nutrition, Osaka, Japan; 4 Department of Respirology, Chibaken Saiseikai Narashino Hospital, Narashino, Japan; 5 Section of Energy Metabolism, Department of Nutrition and Metabolism, National Institute of Biomedical Innovation, Health and Nutrition, Tokyo, Japan; 6 Institute for Protein Research, Osaka University, Osaka, Japan

**Keywords:** Chronic thromboembolic pulmonary hypertension, Gut microbiota, Inflammation, Cytokines, Gut dysbiosis, Inflammatory cytokines

## Abstract

**Background:**

The pathogenesis of chronic thromboembolic pulmonary hypertension (CTEPH) is considered to be associated with chronic inflammation; however, the underlying mechanism remains unclear. Recently, altered gut microbiota were found in patients with pulmonary arterial hypertension (PAH) and in experimental PAH models. The aim of this study was to characterize the gut microbiota in patients with CTEPH and assess the relationship between gut dysbiosis and inflammation in CTEPH.

**Methods:**

In this observational study, fecal samples were collected from 11 patients with CTEPH and 22 healthy participants. The abundance of gut microbiota in these fecal samples was assessed using 16S ribosomal ribonucleic acid (rRNA) gene sequencing. Inflammatory cytokine and endotoxin levels were also assessed in patients with CTEPH and control participants.

**Results:**

The levels of serum tumor necrosis factor-α (TNF-α), interleukin (IL)-6, IL-8, and macrophage inflammatory protein (MIP)-1α were elevated in patients with CTEPH. Plasma endotoxin levels were significantly increased in patients with CTEPH (*P* < 0.001), and were positively correlated with TNF-α, IL-6, IL-8, and MIP-1α levels. The 16S rRNA gene sequencing and the principal coordinate analysis revealed the distinction in the gut microbiota between patients with CTEPH (*P* < 0.01) and control participants as well as the decreased bacterial alpha-diversity in patients with CTEPH. A random forest analysis for predicting the distinction in gut microbiota revealed an accuracy of 80.3%.

**Conclusion:**

The composition of the gut microbiota in patients with CTEPH was distinct from that of healthy participants, which may be associated with the elevated inflammatory cytokines and endotoxins in CTEPH.

**Supplementary Information:**

The online version contains supplementary material available at 10.1186/s12890-022-01932-0.

## Background

Chronic thromboembolic pulmonary hypertension (CTEPH) is a type of pulmonary hypertension (PH) categorized as group 4 PH [1–3]. CTEPH is characterized by the occlusion of the pulmonary arteries with chronic thrombi [1], which leads to the elevation of pulmonary arterial pressure, right heart failure, and death [3]. Specific treatments such as pulmonary endarterectomy, balloon pulmonary angioplasty, and medical treatment for CTEPH have been developed since the 1980s, and advances in the diagnosis and treatment algorithms have contributed to the improved prognosis of CTEPH [4, 5]. Despite these advances, CTEPH is still incurable [2]. Some cases of CTEPH complicated by low cardiac function, persistent PH after specific therapies, renal failure, and malignancy are still at a high risk for mortality [4]. The detailed mechanism of CTEPH development is not fully understood; therefore, research focusing on the pathogenesis of CTEPH is required [3].

Chronic inflammation is conceptually associated with the development of CTEPH [6]. Associated medical conditions, such as inflammatory bowel diseases, splenectomy, and malignancy, can be closely related to pulmonary vascular remodeling and the development of CTEPH [6–8]. Elevated inflammatory cytokines in CTEPH [6, 9] may be associated with its pathogenesis [3], hemodynamics, and prognosis [10, 11]. In chronic organized thrombi in CTEPH, which is a distinct pathological feature compared to the other types of PH [1–3], the accumulation of inflammatory cells, such as lymphocytes, neutrophils, and macrophages [9, 12], are observed. Despite this knowledge, the mechanism of chronic inflammation in CTEPH is not yet fully understood.

Abnormal gut microbiota (dysbiosis) is associated with the development of cardiovascular diseases [13–16]. Gut dysbiosis can lead to translocation of metabolic products and bacterial components from the gut lumen into the bloodstream and can induce an inflammatory processes including activation of macrophages, release of inflammatory cytokines, abnormal aggregation of platelets, and formation of foam cells [13–15]. The induced inflammation can be related to the development of systemic hypertension, atherosclerosis, coronary artery disease, and chronic heart failure [14, 15, 17]. Recent studies suggest that gut dysbiosis appears in experimental animal PH models [18–21] and in patients with pulmonary arterial hypertension (PAH) [22], and may have a causative role in the development of PAH [18, 21]. We hypothesized that gut dysbiosis is also present in CTEPH and is related to the chronic inflammation and pathogenesis of CTEPH.

The aim of this study was to characterize the gut microbiota in patients with CTEPH. In this study, fecal samples were collected from patients with CTEPH and control participants, and the composition of gut microbiota was analyzed by 16S rRNA sequencing. In addition, the relationship between gut dysbiosis and elevated inflammatory cytokines was also assessed.

## Methods

### Patients with CTEPH and control participants

A total of 11 patients diagnosed with CTEPH in Chiba University Hospital were enrolled from October 2017 to January 2020 in this study. Clinical data of patients with CTEPH at diagnosis were analyzed in this study. All of the patients with CTEPH were diagnosed according to the criteria of the 2015 European Society of Cardiology/European Respiratory Society guidelines [23]: 1) mean pulmonary arterial pressure ≥ 25 mmHg; 2) persistent pulmonary embolism confirmed by imaging modalities; 3) symptoms, such as dyspnea and chest pain; and 4) persistent pulmonary embolism lasting > 3 months resisting effective anticoagulation. Hemodynamic data in all patients were assessed using right heart catheterization. The control participants were selected from the database at the National Institutes of Biomedical Innovation, Health and Nutrition and matched with patients with CTEPH in terms of age, sex, and body mass index (BMI) [24].

### Fecal sample collection

Fecal sample collection and deoxyribose nucleic acid (DNA) isolation were performed as previously reported [25]. The fecal samples from patients with CTEPH were collected within 1 week before and after right heart catheterization upon diagnosis at hospital admission, whereas the samples from the control participants were collected in their houses. The time points during the day depended on the participant’s decisions. The fecal samples were placed in tubes containing guanidine thiocyanate solution (Techno-Suruga Laboratory Co., Ltd., Shizuoka, Japan), which were transferred to refrigerators within 5 min after collection. The samples were stored and transported at 4 °C before DNA isolation. DNA was isolated from the fecal samples using the beating method.

### 16S ribosomal ribonucleic acid sequencing

Amplification and sequencing of bacterial 16S rRNA was performed according to our previous reports [25]. The V3-V4 region of the 16S rRNA gene of bacteria was amplified by polymerase chain reaction (PCR) using the following primers: forward, 5′-TCGTCGGCAGCGTCAGATGTGTATAAGCGACAGCCTACGGGNGGCWGCAG-3′, and reverse, 5′-GTCTCGTGGGCTCGGAGATGTGTATAAGAGACAGGACTACHVGGGTATCTAATCC-3′, which was followed by the addition of the sequencing adapters. The amplicons were sequenced using the Illumina MiSeq platform (Illumina Inc., San Diego, CA, USA). A total of 10,000 reads per sample were randomly selected for further analysis. Samples with insufficient read numbers were re-sequenced, and those with repeated insufficient read numbers were excluded.

### Bioinformatics analysis

The obtained paired-end FASTQ data were trimmed and merged before the selection of operational taxonomic units (OTUs). The OTU classification and diversity analyses were performed using the QIIME pipeline (v1.9.1) [26]. All the steps from FASTQ trimming to gut microbiota diversity analysis were automatically performed according to a previously described method [27]. The OTUs were clustered against the SILVA 128 reference database [28] at 97% similarity using the USEARCH algorithm [29]. Taxonomic classification to the genus-level taxa (hereafter referred as genera) was performed using the SILVA 128 reference database. The specific taxonomy names were based on the SILVA database phylogenetic classification standard (https://www.arb-silva.de/browser/ssu/).

### Assessment of plasma endotoxin

Plasma endotoxin levels were measured using a Toxin Sensor Chromogenic LAL Endotoxin Assay Kit (GenScript, Piscataway, NJ, USA) according to the manufacturer’s protocol. Plasma samples were collected from the participants, and standards were incubated with Limulus amebocyte lysate, which was followed by incubation with a chromogenic substrate. Subsequently, the reaction was stopped by the stop solution, and the color-stabilizers were added. Finally, the absorbance at 545 nm was read using a plate reader, and the serum concentration of endotoxin was calculated based on the standard curve.

### Assessment of inflammatory cytokines

Inflammatory cytokines in serum samples were analyzed using the Bio-plex Pro Human Cytokine 27-plex assay (Bio-Rad, Hercules, CA, US), according to the manufacturer’s instructions. A four-fold dilution of serum was incubated with beads and antibodies, and the fluorescence intensity was measured using a Bio-plex system (Bio-Rad). The data were analyzed using Bio-Plex Manager Software ver. 6.1 (Bio-Rad) to calculate the concentration of cytokines based on a standard curve.

### Statistical analysis

The output of the QIIME pipeline in the Biom table format was imported and analyzed using R version 3.5.1 (The R foundation for Statistical Computing, Vienna, Austria). The alpha-diversity indices were calculated using the *estimate_richness* function in the “phyloseq” R-package. The beta-diversity index, calculated by Bray–Curtis distance using genus-level data, was generated using the *vegdist* function in the “vegan” R-package. Principal coordinate analysis (PCoA) was performed using the *dudi.pco* function in the “ade4” R-package. Covariates of gut microbiome β-diversity were identified by calculating the association between continuous or categorical phenotypes and genus-level community coordinate with MANOVA and linear correlations for categorical and continuous variables, respectively. PCoA figures were created using the R package “ggplot2.”

Random forest classification analysis was performed to confirm the potential use of the gut-microbiota community structure as a biomarker to distinguish between patients with CTEPH and the control participants. The *train* function of the “caret” R package was used for random forest classification analysis [30]. The dataset of the classification random forest prediction model was assessed using all data as the training set. In the dataset, descriptors that showed near-zero-variance and absolute correlations of > 0.90 were identified and excluded by calculating the frequency ratio using the *nearZeroVar* function and by creating a correlation matrix using the *findCorrelation* function in the “caret” R package. Finally, descriptors which significantly contributed to the prediction accuracy were selected using the Boruta algorithm [31].

Data were analyzed using statistical software, (GraphPad Prism 9.0.0, GraphPad Software Inc. San Diego, CA, USA). Comparison between the two groups was performed using the Mann–Whitney U-test. The correlation between the two variates was assessed by Spearman’s rank correlation. Data are described as mean ± standard deviation unless otherwise stated. Statistical significance (*P*) was set at < 0.05*.*

### Assessment of dietary habits

Dietary intake was evaluated using a brief self-administered diet history questionnaire (BDHQ), which was developed and validated for use in the Japanese population [32, 33]. The information regarding the dietary habits of all participants were collected around the period when the fecal samples were collected.

## Results

### Characteristics of the participants

The backgrounds of patients with CTEPH and the control paticipants are shown in Table 1. There were no differences in age, sex, and BMI between the control and CTEPH groups because of the matching. One patient had a history of prostate cancer, whereas another patient had a history of Crohn’s disease. None of the participants had implantable medical devices. Eight of the 11 (72.7%) patients with CTEPH had been medically treated with a soluble guanylate stimulator before our diagnosis. All of the 11 patients with CTEPH received anticoagulants at diagnosis (nine patients, warfarin; one patient each rivaroxaban and edoxaban). None of the patients had undergone specific invasive treatments, including pulmonary endarterectomy and pulmonary balloon angioplasty, before the fecal samples were collected. Six (54.5%) patients with CTEPH and two (9.01%) control participants received proton pump inhibitors (PPIs), and the usage was higher in patients with CTEPH (*P* < 0.01). Dietary intake did not differ between the two groups (Additional files 1 and 2).

**Table 1:** Characteristics of patients with CTEPH and control participants

Factor	Group	Control	CTEPH	*P*-value
		(n = 22)	(n = 11)	
Age	Years	65.5 ± 11.6	65.5 ± 12.2	0.94
Sex	Female/male	14-Aug	07-Apr	1
BMI		23.0 ± 4.3	23.7 ± 6.1	0.89
History of acute PE		No data	9	
History of DVT		No data	5	
Disease duration from onset to diagnosis	(months)	No data	52.5 ± 56.9	
NYHA classification	1/2/3/4	No data	0/7/4/0	
Location of thrombus*		No data	Main PA: 6	
			Lobar PA: 4	
			Segmental PA: 1	
Hemodynamics				
Mean PAP	(mmHg)	No data	44.1 ± 9.1	
PAR	(dyne sec cm^−5^)	No data	748 ± 254	
CI	(L/min/m^2^)	No data	2.43 ± 0.51	
Medication				
Proton pump inhibitors		2	6	< 0.01
Antibiotics		0	0	1
Laxatives		0	0	1
Antiflatulents		0	1	0.3
sGC stimulators		0	8	< 0.01

^*^The most proximal site where chronic thromboembolism started within the pulmonary arteries assessed using enhanced computed tomography scan and pulmonary angiography. BMI, body mass index; PE: pulmonary embolism; DVT: deep vein thrombosis; CI, cardiac index; NYHA: New York Heart Association; PA, pulmonary artery; sGC, soluble guanylate cyclase; CTEPH, chronic thromboembolic pulmonary hypertension; PAP, pulmonary arterial pressure; PAR, pulmonary arterial resistance

### Elevated cytokines in patients with CTEPH

**Fig. 1:**
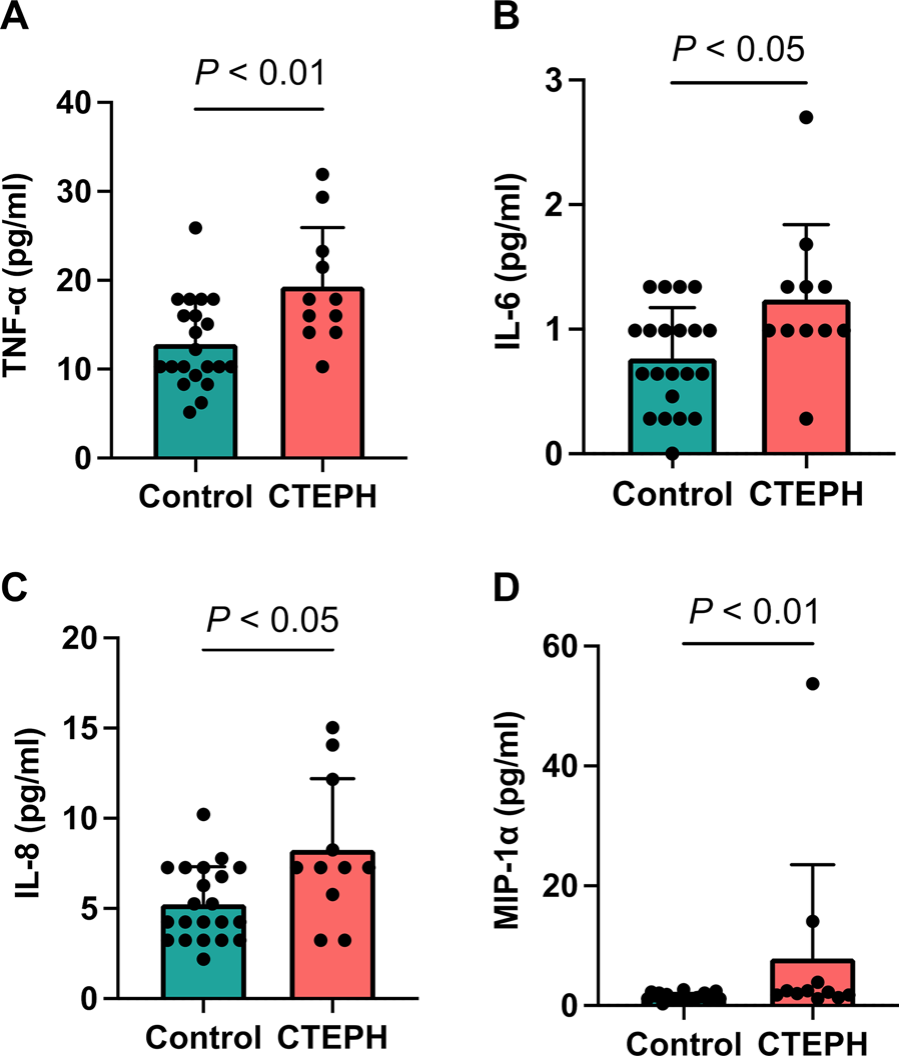
Elevated cytokine levels in chronic thromboembolic pulmonary hypertension and the correlation with serum endotoxin levels. Inflammatory cytokines in the serum are measured using Bio-plex cytokine assays (Bio-Rad). **a** Serum tumor necrosis factor (TNF)-α levels. **b** Serum interleukin (IL)-6 levels. **c** Serum IL-8 levels. **d** Serum macrophage inflammatory protein (MIP)-1α levels

Inflammatory cytokines were first assessed using Bio-plex cytokine assays. Serum tumor necrosis factor (TNF)-α, interleukin (IL)-6, IL-8, and macrophage inflammatory protein (MIP)-1α levels were significantly higher in patients with CTEPH than in the control participants (Figs. 1a–d).

### Elevated endotoxin levels in patients with CTEPH

**Fig. 2:**
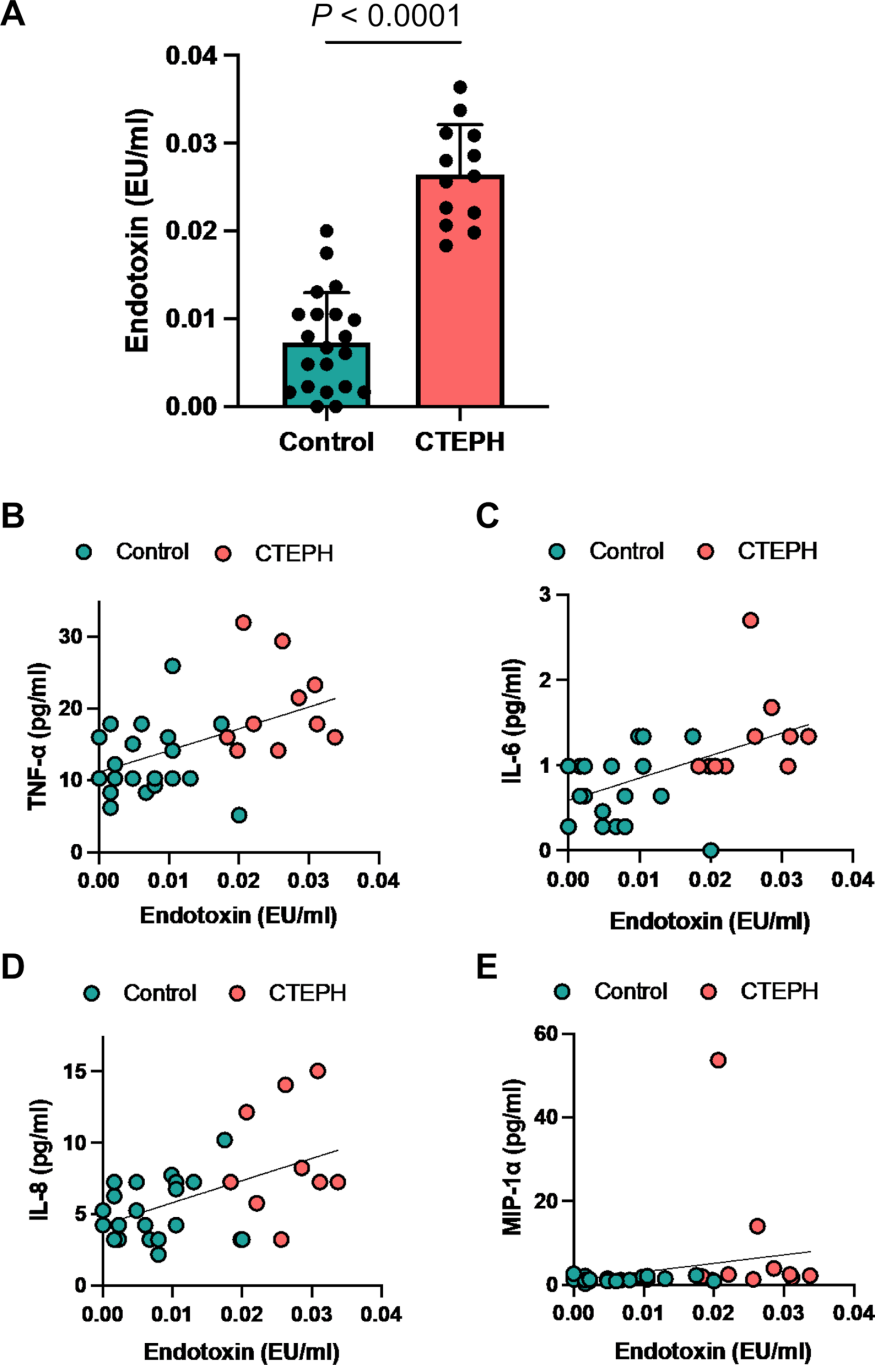
Elevated serum endotoxin level in patients with chronic thromboembolic pulmonary hypertension. **a** Plasma endotoxin levels in the control participants and in patients with CTEPH. **b** to **e** show scatter plots between endotoxin and inflammatory cytokine levels. **b** Tumor necrosis factor (TNF)-α and endotoxin (r = 0.492, *P* < 0.01) levels. **c** Interleukin (IL)-6 and endotoxin (r = 0.565, *P* < 0.01) levels. **d** IL-8 and endotoxin (r = 0.425, *P* < 0.05) levels. **e** Macrophage inflammatory protein (MIP)-1α and endotoxin (r = 0.481, *P* < 0.01) levels

Subsequently, the plasma concentration of endotoxin was assessed in patients with CTEPH and the control participants. Endotoxin is a lipopolysaccharide, a component of the cell membrane of gram-negative bacteria, that can be translocated from the gut lumen to the bloodstream and can induce the expression of inflammatory cytokines and the development of cardiovascular diseases [15]. The endotoxin concentration was significantly higher in patients with CTEPH than in the control participants (*P* < 0.001, Fig. 2a). Serum TNF-α, IL-6, IL-8, and MIP-1α levels were positively correlated with plasma endotoxin levels (TNF-α: r = 0.492, *P* < 0.01; IL-6: r = 0.565, *P* < 0.01; IL-8: r = 0.425, *P* < 0.05; and MIP-1α: r = 0.481, *P* < 0.01, Figs. 2b–e).

### Gut microbiota alteration in patients with CTEPH

**Fig. 3:**
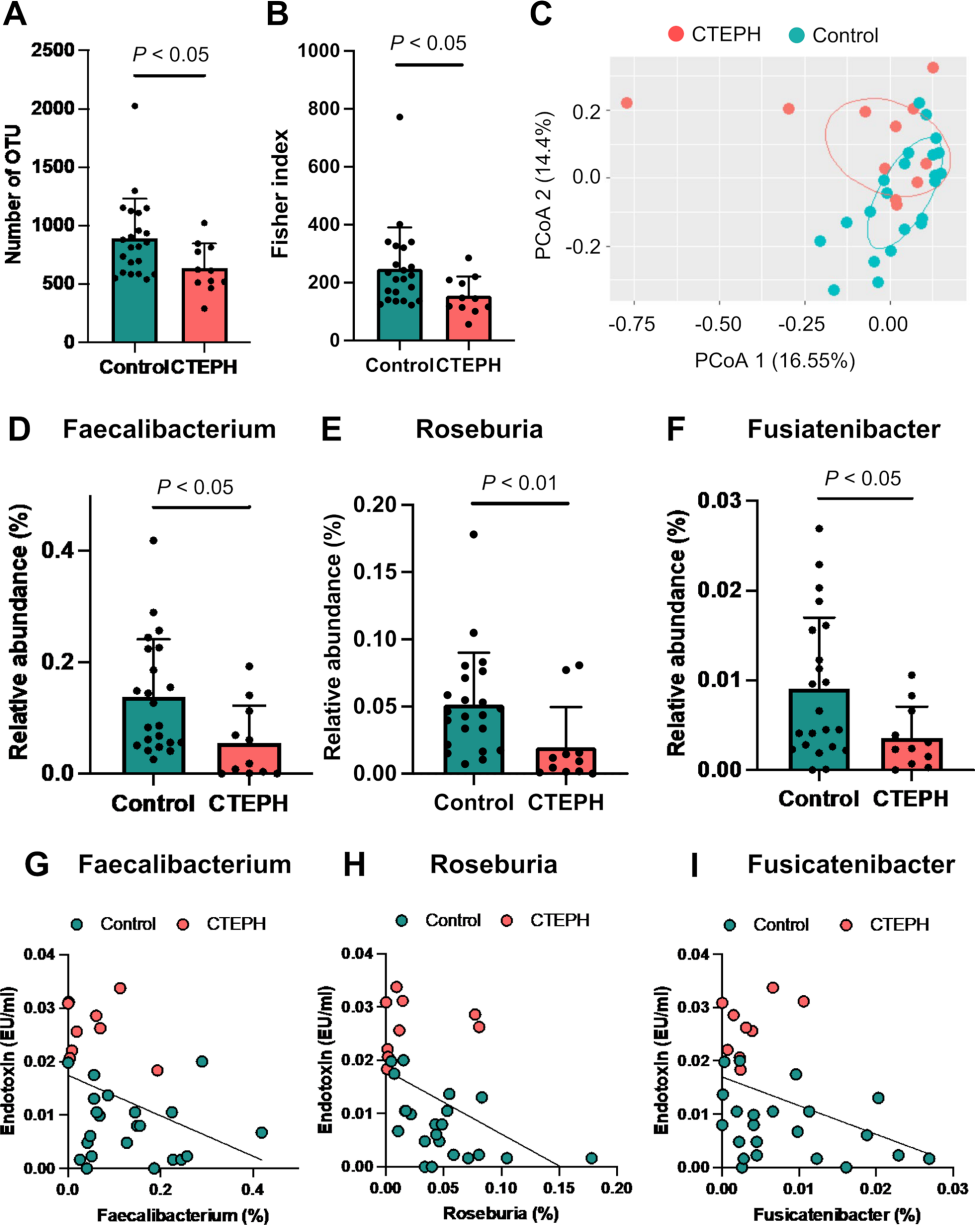
Characteristics of gut microbiota in patients with chronic thromboembolic pulmonary hypertension. **a** and **b** show the alpha-diversity indexes: **a** Observed operational taxonomic unit (OTU) and **b** Fisher index. **c** Principal coordinates analysis (PCoA). **d** to **f** show the relative abundances of butyrate-producing bacteria: **d** *Faecalibacterium*; **f** *Roseburia*; and **f** *Fusicatenibacter*. **g** to **i** show scatter plots between the relative abundance of butyrate-producing bacteria and plasma endotoxin levels. **g** *Faecalibacterium* and endotoxin (r = -0.365, *P* < 0.05) levels. **h** *Roseburia* and endotoxin (r = -0.516, *P* < 0.01) levels. **i** *Fusicatenibacter* and endotoxin (r = -0.397, *P* < 0.05) levels

Subsequently, 16S rRNA sequencing was conducted to assess the composition of the gut microbiota in patients with CTEPH and the control participants. Regarding alpha-diversities, the number of OTUs and the Fisher index were significantly lower in patients with CTEPH than in the control participants (Figs. 3a and b). The PCoA analysis revealed a different composition of gut microbiota in patients with CTEPH than in the control participants (*P* < 0.01, Fig. 3c). *Faecalibacterium*, *Roseburia*, and *Fusicatenibacter* abundance levels were significantly decreased in patients with CTEPH (Figs. 3d–f). The endotoxin concentration was negatively correlated with the relative abundance of these bacteria (*Faecalibacterium*: r = -0.365, *P* < 0.05; *Roseburia*: r = -0.516, *P* < 0.01; and *Fusicatenibacter*: r = -0.397, *P* < 0.05, Figs. 3g–i).

### Random forest analysis

**Fig. 4:**
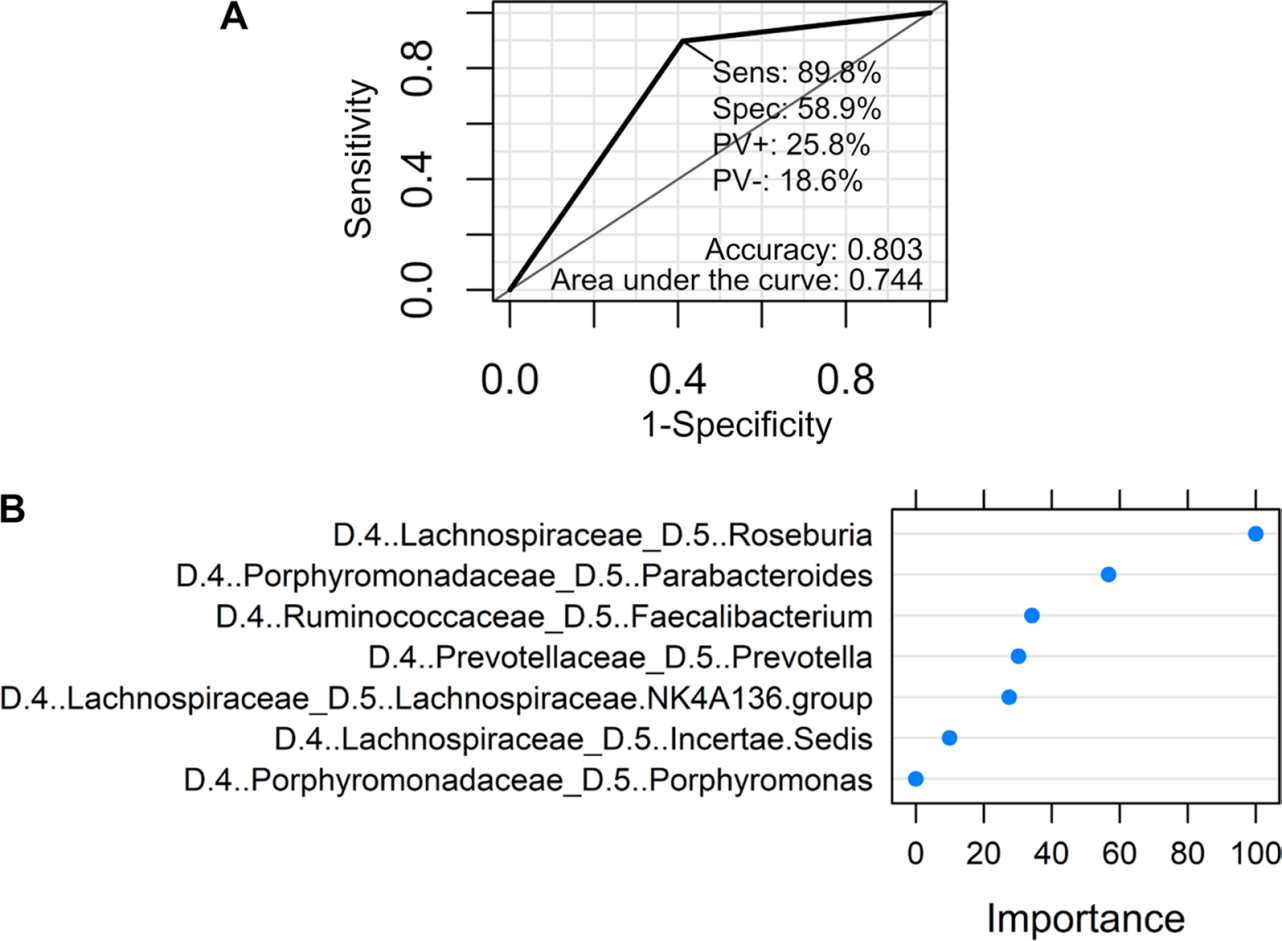
Random forest plot analysis for detecting differences in gut microbiota between the two groups. **a** Receiver operating characteristic (ROC) curve of the random forest model. The area under the curve (AUC) is 0.744, and the accuracy is 0.803. **b** The feature importance of the random forest model

Finally, a prediction model using random forests to distinguish the components of gut microbiota of patients with CTEPH from those of the control participants were created. The detection of the difference in the gut microbiota of the two groups through a classification random forest prediction model had an accuracy of 80.3%, sensitivity of 89.8%, and specificity of 58.9% (Fig. 4a). Bacteria that were affected as notable variables in this predictive model were *Roseburia*, *Parabacteroides*, *Faecalibacterium, Prevotella,* the *Lachnospiraceae* NK4A136 group*,* and *Porphyromonas* bacteria (Fig. 4b).

## Discussion

This study is the first to characterize the gut microbiota of patients with CTEPH. The 16S rRNA sequencing analysis suggested a different composition of gut microbiota in patients with CTEPH compared to that in the control participants and a decreased alpha-diversity in patients with CTEPH; this finding was also supported by a random forest plot demonstrating the distinction with an accuracy of 80.3%. Plasma endotoxin levels were elevated in patients with CTEPH and were positively correlated with the levels of serum inflammatory cytokines, such as TNF-α, IL-6, IL-8, and MIP-1α. Collectively, our results suggest that patients with CTEPH have gut dysbiosis at diagnosis. In addition, the alteration of the gut microbial composition may be associated with the elevation of endotoxin and inflammatory cytokines.

The composition of gut microbiota in patients with CTEPH differed from that of the control participants. Recent studies have suggested that gut dysbiosis occurs in patients with PAH [22] and PH animal models, such as Sugen 5416/hypoxia [20, 21], monocrotaline [19], and hypoxia-induced PH models [18]. To the best of our knowledge, this is the first report to describe the characteristics of the gut microbiota in patients with CTEPH. The PCoA analysis revealed a difference in the composition of the gut microbiota between patients with CTEPH and the control participants, and the results of the random forest analysis supported this distinction. Kim et al. performed random forest analysis to detect the differences in gut microbiota between patients with PAH and control participants and reported an accuracy of 83% [22]. Thus, the analysis of gut microbiota might become a new tool for detecting PH, including CTEPH in the future.

Inflammatory cytokine levels were elevated in patients with CTEPH in this study. It is well known that patients with CTEPH have elevated levels of TNF-α [10, 34], IL-6 [9, 10], IL-8 [9, 35], and MIP-1α [9, 12], which are secreted by the activated macrophages. It has been reported that the accumulation of macrophages is dominant over that of neutrophils in the chronic organized thrombi of patients with CTEPH [36]. TNF-α can affect the coagulation via the elevation of mononuclear tissue factor levels [34] in CTEPH and may be related to the change from acute pulmonary embolism to CTEPH [37]. Von Haehling et al. reported that IL-6 is positively correlated with pulmonary vascular resistance [10]. High serum IL-8 levels have been reported to be related to the development of residual PH after pulmonary endarterectomy [35] and the poor survival of patients with CTEPH [11]. It seems that the macrophages and the cytokines released from macrophages may be associated with the pathogenesis of CTEPH.

Endotoxin levels were significantly higher in patients with CTEPH than in the control participants. Some reports have described elevated endotoxin levels in patients diagnosed with CTEPH [10] and PAH [38] and monocrotaline animal models [38], which supports our results. Endotoxins can translocate from the gut lumen into the bloodstream, which results from an increased intestinal permeability [15, 38]. It is also known that an increase in gut permeability occurs in patients with PAH [25, 26] and an experimental PH model [19]. The endotoxin level is an indirect indicator of increased gut permeability [39]; therefore, elevated endotoxin levels in this study might indicate the increase in gut permeability in patients with CTEPH. Notably, the relative abundances of *Faecalibacterium*, *Roseburia*, and *Fusicatenibacter* were reduced in patients with CTEPH and negatively correlated with the endotoxin levels in these patients and the control participants. *Faecalibacterium*, *Roseburia*, and *Fusicatenibacter* have several biological functions, including anti-inflammatory functions, to maintain intestinal homeostasis. Leclercq et al. reported that the relative abundance of *Faecalibacterium* was negatively correlated with gut permeability [40]. *Roseburia* protects the gut-barrier function via butyrate production and suppresses the intestinal inflammation induced by endotoxin by increasing regulatory T cells and the expression of anti-inflammatory cytokines [41, 42]. A decrease in *Fusicatenibacter* abundance is related to decreased levels of short-chain fatty acids, including butyrate, in patients with liver cirrhosis [43]. As a result of the important variables of the prediction model created through random forest analysis in this study, it was found that there was no association between *Fusicatenibacter* and the gut dysbiosis, but *Faecalibacterium* and *Roseburia* were important variables to characterize patients with CTEPH as in the comparative analysis results. The decrease in bacteria with protective roles for intestinal homeostasis and anti-inflammatory functions in CTEPH may be associated with chronic inflammation via the elevation of endotoxin and cytokine levels.

Endotoxin was positively correlated with inflammatory cytokines in this study. Endotoxin can activate macrophages and endothelial cells via the activation of nuclear factor-kappa B (NFκB) signaling [13, 15, 44, 45]. The activation of NFκB signaling may be associated with the pathogenesis of CTEPH. The NFκB signaling is activated in the endothelial cells within organized thrombi in patients with CTEPH [46], and the expression of inflammatory cytokines is increased [46, 47]. The translocation of endotoxin induced by gut dysbiosis, which has recently been called “metabolic endotoxemia” [48], is associated with chronic low-grade inflammation and the development of cardiovascular diseases [13, 15]. Metabolic endotoxemia may also be related to the pathogenesis of CTEPH.

Although gut dysbiosis in patients with CTEPH was demonstrated, two important issues remain unresolved. The cause of gut dysbiosis in patients with CTEPH is unclear in this study. Dietary habit is an important factor for the alteration of gut microbiota [14, 44]. However, our results regarding the dietary habits suggested that there was no difference in the dietary intake between patients with CTEPH and the control participants and, the effect of diet on gut dysbiosis was limited. PPI treatments can also affect the composition of gut microbiota [49]. More patients with CTEPH used PPI than the control participants in this study; however, the effect on gut dysbiosis could not be evaluated because of the small sample size. Further, this might also have some effects on the gut dysbiosis. In addition, whether gut dysbiosis is a cause or a result of CTEPH remains unclear. It was reported that heart failure and the induced congestion can be related to the alteration of gut microbiota and increased gut permeability [50]. Therefore, it is possible that gut dysbiosis is the result of right heart failure resulting from CTEPH. To solve these issues, it may be helpful to compare patients with CTEPH and acute pulmonary embolism and patients with right heart dysfunction due to CTEPH and other causes.

This study has several limitations. First, the sample size was small. Second, all patients with CTEPH and the control participants in this study were Japanese. Variations in resitential areas and the differences in the dietary habits can affect the characteristics of human gut microbiota [51]; therefore, whether the effect of dysbiosis in patients with CTEPH is global remains unclear. Third, this was an observational study; therefore, it is still unclear whether gut dysbiosis is a cause or a result of CTEPH as described above. Fourth, the time points during the day and the technique of collecting fecal samples during the day depended on the participant’s decision, which might have affected the results. Despite these limitations, we believe that our results suggest the presence of gut dysbiosis in patients with CTEPH and provide important information for understanding the pathogenesis of CTEPH.

## Conclusion

Our findings suggest that patients with CTEPH have gut dysbiosis that can be related to the elevation of endotoxin and inflammatory cytokine levels. The results of this study may provide new perspectives for understanding the pathogenesis of CTEPH.

## Supplementary Information

**Additional file 1.** Dietary intake comparison analysis between patients with CTEPH and control participants. CTEPH: chronic thromboembolic pulmonary hypertansion; FDR: false discovery rate.

**Additional file 2.** Univariate analysis for overall microbiome community variation using the envfit function. FDR: false discovery rate.

## Data Availability

All microbiome next-generation sequencing data files are available from the Sequence Read Archive database (Patients with CTEPH: PRJNA773643, and control participants: DRR247496, DRR247580, DRR247428, DRR247444, DRR247558, DRR247431, DRR247378, DRR247387, DRR247546, DRR247376, DRR247625, DRR247643, DRR247526, DRR247538, DRR247540, DRR247696, DRR248098, DRR247938, DRR247804, DRR247769, DRR247960, DRR247775). The other datasets used in the current study are available from the corresponding author on reasonable request.

## Abbreviations

CTEPH: chronic thromboembolic pulmonary hypertension
PH: pulmonary hypertension
PAH: pulmonary arterial hypertension
BMI: body mass index
DNA: deoxyribose nucleic acid
rRNA: ribosomal ribonucleic acid
PCR: polymerase chain reaction
OTU: operational taxonomic unit
PCoA: principal coordinate analysis
BDHQ: brief self-administered diet history questionnaire
TNF-α: tumor necrosis factor-α
IL: interleukin
MIP: macrophage inflammatory protein
NFκB: nuclear factor-kappa B
PPI: proton pump inhibitor
