# Supplementary material for: Altered gut microbiota and its association with inflammation in patients with chronic thromboembolic pulmonary hypertension: a single-center observational study in Japan
Source: BMC Pulm Med. 2022 Apr 8;22:138. doi: 10.1186/s12890-022-01932-0 (PMC8994357; doi:10.1186/s12890-022-01932-0)
Supplement: Supplementary file 1 — Additional file 1. Dietary intake comparison analysis between patients with CTEPH and control participants. CTEPH: chronic thromboembolic pulmonary hypertansion; FDR: false discovery rate. [file 12890_2022_1932_MOESM1_ESM.pdf]

Additional file 1. Dietary intake comparison analysis between patients with CTEPH and control participants

	Patients with CTEPH				Control participants					
Nutrients	Min.	Med.	Max.	Ave.	Min.	Med.	Max.	Ave.	p-value	FDR
Energy (kcal)	570.35	1447.38	2055.65	1372.42	1061.47	1710.72	2474.82	1754.83	0.07	0.71
Energy from total protein (%energy)	12.95	16.01	21.27	16.53	12.21	17.61	23.73	17.30	0.74	0.91
Energy from animal protein (%energy)	6.71	8.62	13.26	9.67	5.84	10.98	16.84	10.64	0.59	0.87
Energy from vegetable protein (%energy)	5.08	6.53	9.15	6.86	5.28	6.60	8.49	6.65	0.91	0.97
Energy from total fat (%energy)	19.97	26.03	40.48	27.59	16.96	30.43	38.31	29.21	0.47	0.87
Energy from animal fat (%energy)	9.05	13.50	20.29	14.36	8.73	14.62	22.19	14.99	0.65	0.88
Energy from vegetable fat (%energy)	8.54	11.84	20.62	13.23	4.21	13.49	20.19	14.22	0.37	0.87
Energy from saturated fat (%energy)	4.60	6.52	11.46	7.48	4.95	8.16	10.53	7.83	0.74	0.91
Monounsaturated fat (%energy)	6.43	9.38	14.92	9.76	5.80	9.90	14.00	10.16	0.59	0.87
Polyunsaturated fat (%energy)	4.03	6.40	10.69	6.48	3.10	7.70	10.03	7.22	0.33	0.87
Energy from carbohydrate (%energy)	39.97	58.69	65.72	55.88	39.94	51.84	69.66	53.49	0.4	0.87
Alcohol (%energy)	0.00	0.00	11.38	1.38	0.00	0.52	9.27	2.02	0.06	0.79
Ash content per 1000 kcal (g)	9.24	10.63	17.25	11.29	6.99	11.55	15.51	11.49	0.56	0.87
Sodium per 1000 kcal (mg)	2131.70	2497.60	4065.01	2635.92	1558.06	2618.23	3307.45	2486.93	0.98	0.98
Potassium per 1000 kcal (mg)	931.71	1516.21	2272.92	1501.71	858.40	1639.42	2713.94	1711.93	0.23	0.87
Calcium per 1000 kcal (mg)	175.98	326.35	537.93	330.52	165.99	362.78	673.48	384.00	0.31	0.87
Magnesium per 1000 kcal (mg)	105.64	141.09	216.35	146.45	96.41	163.88	256.51	163.41	0.23	0.87
Phosphorus per 1000 kcal (mg)	493.48	604.14	820.64	629.66	414.63	666.98	991.91	675.89	0.53	0.87
Iron per 1000 kcal (mg)	2.39	4.73	8.18	4.80	2.88	5.35	8.60	5.21	0.27	0.87
Zinc per 1000 kcal (mg)	3.51	4.62	5.48	4.63	3.93	4.77	6.78	4.91	0.45	0.87
Copper per 1000 kcal (mg)	0.53	0.62	0.93	0.66	0.55	0.65	0.92	0.67	0.56	0.87
Manganese per 1000 kcal (mg)	1.36	1.87	5.72	2.33	1.25	1.80	2.94	1.95	0.68	0.9
Retinol per 1000 kcal (μg)	114.07	165.35	452.96	207.57	65.17	255.65	529.70	273.81	0.29	0.87
β-Carotene equivalents per 1000 kcal (μg)	621.87	1766.34	3359.01	1874.32	561.84	2895.44	5858.81	3119.03	0.06	0.79
Retinol equivalents per 1000 kcal (μg)	183.61	376.96	531.35	365.96	197.62	524.63	952.77	535.78	0.03	0.79
Vitamine D per 1000 kcal (μg)	4.30	8.12	23.67	10.01	2.23	10.14	25.59	10.54	0.88	0.97
α-Tocopherol per 1000 kcal (mg)	2.26	3.87	6.79	4.13	1.78	5.19	8.06	4.88	0.09	0.84
Vitamine K per 1000 kcal (μg)	28.99	166.11	288.09	162.10	64.78	210.39	406.95	220.49	0.2	0.87
Vitamine B1 per 1000 kcal (mg)	0.36	0.46	0.62	0.47	0.33	0.51	0.78	0.50	0.65	0.88
Vitamine B2 per 1000 kcal (mg)	0.61	0.84	1.32	0.84	0.55	0.80	1.34	0.85	0.91	0.97
Niacin per 1000 kcal (mg)	6.07	8.81	17.64	10.04	7.18	10.38	16.32	11.08	0.23	0.87
Vitamine B6 per 1000 kcal (mg)	0.44	0.73	1.40	0.76	0.49	0.78	1.22	0.82	0.29	0.87
Vitamine B12 per 1000 kcal (μg)	2.17	4.99	11.91	5.88	2.27	5.77	13.95	6.42	0.84	0.96
Folic acid per 1000 kcal (μg)	110.55	206.09	518.88	225.58	103.16	220.82	455.16	241.92	0.35	0.87
Pantothenic acid per 1000 kcal (mg)	3.00	3.74	4.53	3.82	2.84	3.79	5.90	4.00	0.59	0.87
Vitamine C per 1000 kcal (mg)	48.59	78.92	193.72	87.46	19.27	75.96	150.55	79.15	0.81	0.95
Saturated fatty acids per 1000 kcal (g)	5.11	7.24	12.74	8.31	5.50	9.07	11.70	8.70	0.74	0.91
Monounsaturated fatty acids per 1000 kcal (g)	7.15	10.42	16.58	10.85	6.44	11.00	15.56	11.29	0.59	0.87
Polyunsaturated fatty acids per 1000 kcal (g)	4.48	7.11	11.87	7.20	3.45	8.55	11.14	8.02	0.33	0.87
Cholesterol per 1000 kcal (mg)	121.84	231.49	308.36	232.73	118.76	227.33	419.97	241.65	0.98	0.98
Water soluble dietary fiber per 1000 kcal (g)	0.94	1.88	3.02	1.84	0.57	1.94	3.36	1.99	0.56	0.87
Insoluble dietary fiber per 1000 kcal (g)	3.48	5.34	9.21	5.35	2.84	5.57	8.80	5.66	0.47	0.87
Total dietary fiber per 1000 kcal (g)	4.58	7.33	12.23	7.37	3.72	8.07	12.41	7.94	0.53	0.87
Salt equivalent per 1000 kcal (g)	5.39	6.33	10.14	6.65	3.92	6.61	8.33	6.27	0.98	0.98
Sugar per 1000 kcal (g)	0.00	4.93	9.94	4.92	0.31	5.51	15.80	6.17	0.47	0.87
Alcohol per 1000 kcal (g)	0.00	0.00	16.25	1.98	0.00	0.74	13.24	2.89	0.06	0.79
Daidzein per 1000 kcal (mg)	1.73	6.47	17.70	8.32	2.50	7.77	22.14	8.75	0.62	0.88
Genistein per 1000 kcal (mg)	2.98	11.09	30.52	14.16	4.22	13.25	37.95	14.89	0.59	0.87
ω:3 polyunsaturated fatty acids per 1000 kcal (g)	0.59	1.47	2.61	1.65	0.46	1.82	2.49	1.71	0.81	0.95
ω:6 polyunsaturated fatty acids per 1000 kcal (g)	3.84	5.51	9.20	5.51	2.97	6.65	9.31	6.28	0.35	0.87

CTEPH: chronic thromboembolic pulmonary hypertansion; FDR: false discovery rate.
